# Design of the PROUD study: PCR faeces testing in outpatients with diarrhoea

**DOI:** 10.1186/s12879-016-1371-z

**Published:** 2016-01-30

**Authors:** Alwin Schierenberg, Martine D. Nipshagen, Berna D. L. Broekhuizen, Alma C. van de Pol, Patricia C. J. Bruijning-Verhagen, Johannes G. Kusters, Rob Schuurman, Sanne van Delft, Marie-Josée J. Mangen, Niek J. de Wit, Marc J. M. Bonten

**Affiliations:** 1 Julius Center for Health Sciences and Primary Care, University Medical Center Utrecht, Universiteitsweg 100, P.O. Box 85500, 3584 CG Utrecht, The Netherlands; 2 Department of Medical Microbiology, University Medical Center Utrecht, Heidelberglaan 100, 3584 CX Utrecht, The Netherlands; 3 Saltro Diagnostic Center, Mississippidreef 83, 3565 CE Utrecht, The Netherlands

**Keywords:** PCR, Molecular diagnostics, Gastroenteritis, Infectious intestinal disease, Diarrhoea enteropathogens, Primary care, General practitioner, Faeces testing, Economic evaluation

## Abstract

**Background:**

Infectious intestinal disease (IID) is an important cause of morbidity in developed countries and a frequent reason for general practitioner (GP) consultation. In recent years polymerase chain reaction (PCR) based techniques have gradually replaced conventional enteropathogen detection techniques like microscopy and culture in primary care patients suspected of IID. PCR features testing of multiple enteropathogens in a single faecal sample with shorter turnaround times and greater sensitivity compared to conventional techniques. However, the associated costs and benefits have not been quantified. Furthermore, primary care incidence and prevalence estimates of enteropathogens associated with IID are sparsely available and predominantly based on conventional techniques. The PROUD-study (PCR diagnostics in Outpatients with Diarrhoea) determines: 1) health (care) effects and 2) cost-effectiveness of PCR introduction in primary care patients suspected of IID; 3) occurrence of major enteropathogens in primary care patients suspected of IID.

**Methods:**

A before-after cohort study will be performed of patients with suspected IID consulting a GP in the Utrecht General Practitioner Network (UGPN), covering the before period (2010–2011) with conventional testing and the after period (2013–2014) with PCR testing. Prospective study data on patient characteristics and primary outcome measures (i.e. healthcare use and disease outcome) will be collected from electronic patient and laboratory records in 2015 and 2016. The effect of PCR introduction is investigated by comparing the primary outcome measures and their associated healthcare costs between the conventional period and the PCR period, and is followed by a cost-effectiveness analysis. To determine the occurrence of enteropathogens associated with IID in primary care, routine care faeces samples from the year 2014 will be screened using PCR.

**Discussion:**

The PROUD-study will quantify the costs and effects of the introduction of PCR techniques for enteropathogens in primary care patients suspected of IID and generate up-to-date and sensitive estimates of enteropathogen occurrence among primary care patients.

**Electronic supplementary material:**

The online version of this article (doi:10.1186/s12879-016-1371-z) contains supplementary material, which is available to authorized users.

## Background

Despite high hygienic standards and socioeconomic level, infectious intestinal disease (IID) remains a major cause of morbidity in developed countries, with a reported incidence of 19–83 cases/100 person years [1–5]. The direct healthcare costs for all cause gastroenteritis in the Netherlands have almost doubled in the last decade and are estimated at €147 million per year [6].

In primary care IID is among the most frequent reasons for consultation [7], but generally requires supportive treatment only as most IID episodes are self-limiting. According to Dutch guidelines, microbiological faeces testing to detect the causative pathogen is only recommended for high-risk patients that may require antimicrobial treatment or pose a substantial transmission risk to others, such as healthcare or food-production workers.

In recent years, molecular based faeces testing using Polymerase Chain Reaction (PCR) techniques have become available for primary care use, replacing conventional microbiological diagnostic techniques like culture and microscopy. PCR faeces testing allows detection of multiple enteropathogens in a single sample with shorter turnaround times and greater sensitivity compared to conventional methods [8, 9]. Implementation of PCR initially requires a substantial investment, but can potentially lead to an overall cost reduction by extensive automation. Due to its potential added clinical value, primary care diagnostic laboratories in the Netherlands have increasingly replaced conventional techniques by PCR testing. To what extent the introduction of PCR in primary care has affected detection rates of causative enteropathogens, disease outcome and the use of healthcare resources, such as antibiotic prescribing and faeces testing has not yet been determined. Before further implementation of PCR faeces testing in primary care can be recommended, it is crucial to identify its effects and to evaluate if PCR faeces testing in patients with suspected IID is cost-effective in comparison to conventional testing.

Here the study design and rationale of the PROUD-study (PcR faeces testing in OUtpatients with Diarrhoea), a primary care based study on IID and PCR introduction in the Netherlands, is described.

## Methods

### Objectives

The primary objectives of the PROUD study are:

1. To determine the effects of PCR introduction for enteropathogen detection in primary care on important aspects of *healthcare use* among patients consulting for suspected IID, including the rate of microbiological faeces testing, (antibiotic) drug prescription, reconsultation and referral to medical specialist, and on their *disease outcome*, including IID duration and confirmed enteropathogens.
2. To determine the cost-effectiveness of PCR faeces testing in primary care in comparison to conventional testing taking both a program perspective, including only testing costs, and a healthcare payer perspective, including both testing costs and other direct healthcare costs.
3. To determine the occurrence of major enteropathogens as detected by PCR testing among primary care patients with suspected IID.

Secondary objectives investigated in the PROUD study are outlined in Additional file 1.

### Study design

To evaluate the introduction of PCR faeces testing and improve clinical management of IID in primary care, a before-after cohort study will be performed, including prospective data of a 2-year ‘before‘period with conventional testing and a 2-year ‘after’ period with PCR testing, and excluding a one-year wash-in period in which PCR testing is introduced. A before-after study design is adopted while the novel diagnostic technique (PCR) is already implemented in the region of our institute. Besides this reason, a prospective randomized design would require substantial human and financial resources in order to recruit sufficient patients to study the primary objectives. The main advantage of a before-after study design is that it prevents interference with usual clinical care (i.e. GPs are not aware of the on-going study) and laboratory logistics, and the use of prospective study data, therefore representing routine clinical practice.

To determine primary care occurrence of major enteropathogens as detected by PCR faeces testing, a nested 1-year microbiological study with full panel enteropathogens PCR testing for IID causing bacteria, parasites and viruses, will be performed.

### Study population

The study population includes patients registered with a general practice affiliated with both the Utrecht General Practitioner Network (UGPN) and Saltro Diagnostic Center. The UGPN database contains pseudonymous routine healthcare data extracted from the Electronic Medical Records (EMR) of 225 GPs in metropolitan Utrecht with approximately 330,000 patients enlisted (in 2013). The general practices contributing to the database contain a representative sample of the Dutch population. The GPs working in participating practices are trained in correct use of International Classification of Primary Care (ICPC) coding and have on average 10 years’ experience in systematic coding of disease episodes [10]. Saltro Diagnostic Center is a large primary care laboratory operating in the UGPN service area and replaced conventional enteropathogen testing by PCR in April 2012.

**Fig. 1:**
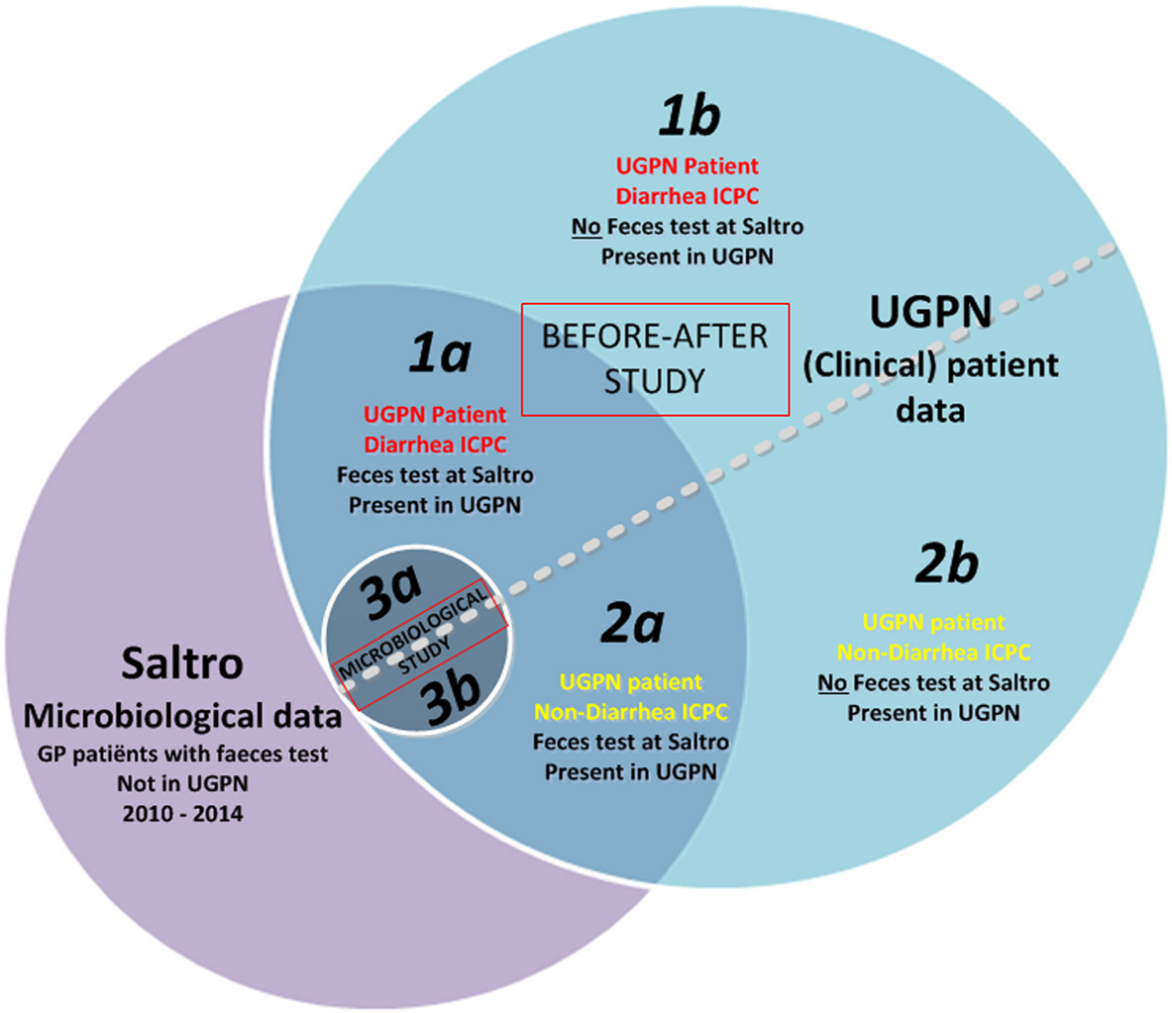
Sources and composition of study population of ‘before’ and ‘after’ cohorts. Blue circle: Clinical patient data from Utrecht General Practitioner Network (UGPN) of 2010–2014. Purple circle: faeces testing result from Saltro Diagnostic Center of 2010–2014. 1/2a: UGPN patient with a faeces test. 1/2b: UGPN patient without faeces test. 1a/b: UGPN patients with a coded episode of suspected Infectious Intestinal Disease (IID). 2a/b: UGPN patients without a coded episode of suspected IID. 3a/b: Patients included in microbiological study with/without a coded episode of suspected IID

In the before-after study, subjects eligible for inclusion are patients consulting a UGPN practitioner with suspected IID in the before period (2010–2011) or after period (2013–2014) (Fig. 1, population 1a and 1b). Suspected IID is defined as a consultation coded with ICPC D11 (diarrhoea), D70 (gastrointestinal infection) or D73 (suspected infectious gastroenteritis). A one-year wash-in period (2012) is excluded from the analysis to account for adaptation to the new PCR strategy.

In the microbiological study, all patients referred by an UGPN physician to Saltro Diagnostic Center for microbiological faeces testing in 2014 are included (Fig. 1, population 3a and 3b). Therefore, the microbiological study population includes all PCR tested UGPN patients, regardless of the assigned ICPC codes.

### Measurements before-after study

#### Clinical patient data

For each patient with an episode of suspected IID, routine care data on *patient demographics*, *healthcare use* and *disease outcome* are extracted from the EMR. *Patient demographics* include age, gender, ICPC coded co-morbidities, immunocompromised status (e.g. chronic immunosuppressive therapy, chronic renal and/or liver disease, current malignancy and chemotherapy) and other assumed IID risk factors present at the first time of consultation for suspected IID (Additional file 2). *Healthcare use* per disease episode (defined as a period between the first and last consultation for the same indication with a minimum of 60 days) includes drug prescriptions, assigned ICPC codes (Additional file 3), and the number and type of consultations per episode. *Disease outcome* includes duration per episode and enteropathogens identified. Mortality is not measured, as it is not part of the UGPN data and not retrievable via Municipal Administration (GBA), since direct identification of included subject is not possible.

#### Linkage of clinical patient data with faeces test data

To link (clinical) patient data with corresponding microbiological test results, all patients identified in the UGPN database are linked with the laboratory records from Saltro Diagnostic Center by a ‘trusted third party’ using a pseudonimization procedure in accordance with the Dutch Health Insurance Portability and Accountability Act of 1996.

#### Faeces testing

Results for 14 enteropathogens (Table 1) of patients with suspected IID who underwent conventional faeces testing (microscopy, culture and/or enzyme immunoassay [EIA]) in the before period and with primarily PCR testing in the after period, are gathered. A routine culture attempt is performed on all positive bacterial PCR tests. In principle, this allows us to obtain the corresponding isolates and to perform further serologic, relevant phenotypic or genetic typing. For both methods the relative sensitivity, specificity and efficiency will be determined, also proving a basis for the cost-effectiveness analysis (objective 2).

**Table 1:** IID causing enteropathogens (*n* = 14) included in “before-after” study

Enteropathogen	Before test		After test	
	Test method	Identification method	Test method	Identification method
*Campylobacter* spp.	Culture	Campylobacter selective agar, hippurate hydrolysis identification	PCR	LightMix Modular Gastro Bacteria
*Clostridium difficile*	EIA	ImmunoCard Toxine A/B	EIA	ImmunoCard Toxine A/B
*Salmonella* spp.	Culture	XLD agar, Vitek identification	PCR	TIB MOLBIOL LightMix Modular Gastro Bacteria
*Shigella* spp.	Culture	XLD agar, Vitek identification	PCR	TIB MOLBIOL LightMix Modular Gastro Bacteria
*Plesiomonas* spp	Culture	XLD agar, Vitek identification	PCR	TIB MOLBIOL LightMix Modular Gastro Bacteria
*Yersinia* spp.	Culture	CIN agar, Vitek identification	PCR	TIB MOLBIOL LightMix Modular Gastro Bacteria
*Blastocystis hominis*	Microscopy	Direct with accumulation (Ridley)	PCR	TIB MOLBIOL LightMix Modular Gastro Parasites
*Cryptosporidium* spp.	Microscopy	Direct with accumulation (Ridley)	PCR	TIB MOLBIOL LightMix Modular Gastro Parasites
*Dientamoeba fragilis*	Microscopy	TFT	PCR	TIB MOLBIOL LightMix Modular Gastro Parasites
*Entamoeba histolytica*	Microscopy	Direct with accumulation (Ridley)	PCR	TIB MOLBIOL LightMix Modular Gastro Parasites
*Giardia* spp.	Microscopy	Direct with accumulation (Ridley)	PCR	TIB MOLBIOL LightMix Modular Gastro Parasites
Adenovirus 40/41	ICS	R-Biopharm RIDA Quick Adeno/Rotavirus	ICS	R-Biopharm RIDA Quick Adeno/Rotavirus
Norovirus	ICS	R-Biopharm RIDA Quick Nonvirus	ICS	R-Biopharm RIDA Quick Nonvirus
Rotavirus	ICS	R-Biopharm RIDA Quick Adeno/Rotavirus	ICS	R-Biopharm RIDA Quick Adeno/Rotavirus

*CIN* cefsulodin-irgasan-novobiocin, *EIA* enzyme immunoassay, *ICS* immunochromatographic strip, *TFT* triple faeces test, *XLD* xylose-lysine-deoxycholate

### Measurements for microbiological study

For the microbiological study (objective 3), faecal samples sent for microbiological testing from all participating UGPN practices to Saltro Diagnostic Center in 2014 are tested with PCR (Additional file 4) for 19 of the enteropathogens as described in Table 2.

**Table 2:** IID causing enteropathogens (*n* = 19) included in PCR testing for microbiological study

Enteropathogen	PCR assay	PCR system	Positive test
*Campylobacter* spp., *Salmonella spp., Shigella spp., Plesiomonas spp., Yersinia spp.*	TIB MOLBIOL LightMix® Modular Gastro Bacteria	LightCycler 480 II	Ct <45
*Clostridium difficile*	R-Biopharm RIDA®GENE Clostridium difficile Toxin A/B	LightCycler 480 II	Ct <45
EHEC/STEC, EPEC	R-Biopharm RIDA®GENE E.Coli Stool Panel 1	LightCycler 480 II	Ct <45
*Blastocystis hominis, Cryptosporidium spp., Dientamoeba fragilis, Entamoeba histolytica, Giardia spp.*	TIB MOLBIOL LightMix® Modular Gastro Parasites	LightCycler 480 II	Ct <45
Adenovirus 40/41, Astrovirus, Norovirus, Rotavirus, Sapovirus	Laboratory Developed Test	ABI 7500	Ct <45

*EHEC* enterohaemorrhagic *Escherichia coli*, *STEC* shiga toxin-producing *Escherichia coli*, *EPEC* enteropathogenic *Escherichia coli*

### Outcome measures

In the before-after study the outcome measures are: *healthcare use*; operationalized as the proportions of faeces testing, (antibiotic) drug prescribing, number of GP consultations per disease episode and specialist referrals during each period among patients consulting their GP for suspected IID, and *disease outcome*; operationalized as confirmed IID indicated by a positive test result and disease duration defined as the number of days between the first and the last consultation of the episode (objective 1).

In the economic evaluation, several outcome measures for *costs* and *effects* are included. For *costs* healthcare costs, testing costs and total costs (healthcare and testing costs) per episode and in total, are included. Included *effects* per disease episode are the proportion of faeces testing, detected relevant enteropathogens, antibiotic prescription, reconsultation and referral in the before and after periods. To compare conventional testing to PCR testing (objective 2), the difference in *costs* will be compared to a difference in the mentioned *effects* and expressed as a cost-effectiveness ratio (CER), for example the additional costs per detected relevant enteropathogen.

In the microbiological study, the outcome measure is the absence or presence per enteropathogen in the collected faeces samples detected by PCR (objective 3).

### Statistical analysis

#### Objective 1

To estimate the effect of PCR introduction on *healthcare use* and *disease outcome* the above mentioned outcome measures are compared between the patient cohorts in the before and after period, taking into account potential differences in patient characteristics and comorbidities between the two cohorts (Additional file 2). Differences in continuous and categorical outcome measures are quantified by Mann–Whitney U tests and Fisher’s exact tests, respectively. To assess the independent effect of the introduction of PCR on the proposed outcome measures, an interrupted time series analysis is performed incorporating potential confounding variables including age, gender, policy deductibles of health care insurance and patient co-morbidities (DM, COPD, asthma, cardiovascular diseases, inflammatory bowel disease [IBD], irritable bowel syndrome [IBS], and immunocompromising disease and medication). This type of analysis uses segmented regression to measure changes in level and slope in the before period compared to the after period to control for secular trends in the data [11], making adjustment for individual-level characteristics unnecessary [12].

#### Objective 2

Healthcare costs are calculated by multiplying the extracted healthcare resources used with their unit cost prices according to the Dutch guidelines [13, 14]. Test costs are calculated based on the unit cost prices according to the Dutch Healthcare Authority (NZA) tariffs, material and overhead costs for conventional and PCR testing. All costs are expressed for the year 2015 and considered both undiscounted and discounted (i.e. 4 %). To determine the cost-effectiveness of PCR testing compared to conventional faeces testing, a cost-effectiveness analysis (CEA) is performed from both a program perspective (i.e. testing costs only) and from a healthcare payer perspective (i.e. healthcare costs and testing costs) respectively, and expressed as CERs. Subgroup analysis is performed for various age groups and for all pathogen-groups (i.e. bacteria, parasites and virus testing). Finally, cost-effectiveness for scenarios with different levels for PCR costs and cut-off values for PCR sensitivity (Ct-values) are evaluated. Adjustment for potential confounding variables is performed as described above. It is assumed that within the study periods no differences in healthcare setting, like changes in personnel (e.g. GP) and background changes in GP population, occur that significantly influence the results.

#### Objective 3

To determine the incidence of enteropathogens in primary care patients suspected of IID as detected by PCR testing, the proportion and 95 % CI of individual and combined infections are calculated. First using the number of patients with microbiological testing performed as the denominator, and secondly by extrapolation to the general population of patients that visit the GP with suspected IID through standardization by age, gender, patient comorbidities (as described under objective 1) and episode ICPC code according to the distribution in the complete cohort of 2014.

### Power calculations

We assessed the statistical power of before-after study, covering a 2-year “before” period with conventional testing and a 2-year “after” period with PCR testing and excluding a one-year wash-in period in between. These calculations were based on the minimal detectable difference between the two study periods for two clinically important outcomes: the proportions of IID patients in whom diagnostic testing is performed and the proportion of prescribed antibiotics. Two-tailed Fisher’s exact tests were performed using a significance level of 5 % and a power of 90 %. Based on previous evidence it is anticipated that between 4,615 and 11,539 of the 330,000 UGPN patients annually consulted their GP with suspected IID (Fig. 1, population 1a/b) [1], of which around 12 % (554–1,385) are tested for enteropathogens in the before period (Fig. 1, population 3a) [15]. The minimal detectable difference in proportion of diagnostic tests performed between the two periods ranges between 1 to 1.6 % depending on the actual number of suspected IID cases. Anticipating a 27 % antibiotic prescription rate among patients with suspected IID in the conventional period, around 9,230–23,078 patients per year were expected to receive an antibiotic prescription [15]. Depending on the actual number of suspected IID cases, the minimal detectable difference in antibiotic prescribing rates ranged between 1.3 and 2.1 %.

For the one-year microbiological study with PCR testing, the achievable precision in estimates for enteropathogen proportions was evaluated through exploration of the 95 % confidence interval (CI) widths over a range of plausible enteropathogen proportions. As described above we expected at least 554–1,385 UGPN patients per year with an episode of suspected IID and a faeces test. The width of the CI ranged between ±0.2 % for the least expected pathogen (*Shigella* spp.) and based on the largest estimated sample size, to ±3.4 % for the most prevalent pathogen (*Blastocystis hominis*) and smallest sample size (Additional file 5).

It was concluded that a before-after study including two periods of 2 years and microbiological study including a 1-year period of PCR testing were sufficient to study our primary objectives.

### Ethical approval

The act on medical research involving human subjects does not apply to this study and therefore official approval of this study by the Institutional Review Board (IRB) of the University Medical Center Utrecht was not required (IRB-number: 13–480).

## Discussion

The PROUD study will quantify the effects of the introduction of PCR faeces testing and its cost-effectiveness in primary care patients with suspected IID, and may guide further large-scale implementation of PCR testing in primary care. It will also describe the epidemiology of IID and aetiology of enteropathogens detected with PCR, both relevant for clinical practice and healthcare policy making.

Conventional and molecular techniques for the detection of enteropathogens exhibit different test characteristics, where PCR-based testing has a lower turnaround time and its increased sensitivity may yield 1.4 to 3-fold higher detection rate [8, 9, 16, 17]. The latter leads to a higher diagnostic yield, but may also detect non-relevant microorganisms. It is therefore important to include the effects of the test results on patient management in the evaluation of introducing PCR-based testing, rather than exclusively focusing on the technical performance of these diagnostic techniques.

### Strengths

The use of routine study data and a before-after study design prevents interference with routine clinical care in primary care patients with suspected IID, excluding bias introduced by an observer effect [18]. However, the before-after study design may be susceptible to bias, but has previously been used successfully to evaluate the cost-effectiveness of an infection control program to reduce nosocomial respiratory syncytial virus transmission and an intervention to reduce the spread of influenza during the H1N1 pandemic [19, 20].

The possibility to use the UGPN database, including 330,000 patients, allows us to study sufficient numbers of patients in a relatively short time period. Naturally, our study domain is restricted to patients consulting their GP for IID, which we consider the relevant population this research question. Lastly, the use of a third trusted party enables merging of patient data on an individual level and ensures the anonymity of included patients.

### Limitations

Ideally a randomised controlled trial comparing effects between two diagnostic strategies without extraneous (other than the diagnostic strategy) factors would have been performed. However, a prospective randomised design would require substantial human and financial resources in order to recruit sufficient patients to study the primary objectives and exceeds the available budget. Moreover, as PCR faeces testing was already implemented in routine practice when initiating the PROUD study, a comparison to conventional techniques was logistically unfeasible.

In the economic evaluation, a restricted perspective has to be taken, as we only have information on testing costs and potential savings in healthcare costs in primary care. Potential positive monetary and health effects due to, for example reduced hospitalization, earlier detection of an outbreak leading to reduced monitoring costs and the prevention of disease complications (e.g. sepsis, sequelae) through timely diagnosis and appropriated treatment were not included as this information is lacking in the consulted databases. Therefore, this economic evaluation will lead to conservative CER estimates, but will resemble the costs and effects that are relevant for the primary care domain.

Furthermore, a one-year microbiological study will potentially be more prone to fluctuations when compared to studies including multiple years. Yet, yearly variation is mainly observed for enteric viruses, whereas for more clinically relevant bacterial and parasitic enteropathogens fluctuations are less common.

## Additional files

Additional file 1: **Secondary objectives of the PROUD study.** (DOCX 18 kb)

Additional file 2: **Measurements of basic patient characteristics and comorbidities coded with International Classification of Primary Care (ICPC).** (DOCX 16 kb)

Additional file 3: **Inclusion list of medication coded with Anatomical Therapeutic Chemical (ATC) classification system.** (DOCX 15 kb)

Additional file 4: **PCR laboratory procedures in microbiological study.** (DOCX 17 kb)

Additional file 5: **Expected 95 % CIs for enteropathogen proportions in a 1-year microbiological study.** (DOCX 24 kb)

## Abbreviations

ATC: Anatomical therapeutic chemical classification system
CEA: cost-effectiveness analysis
CER: cost-effectiveness ratio
CI: confidence interval
CIN: cefsulodin-irgasan-novobiocin
COPD: chronic obstructive pulmonary disease
DM: diabetes mellitus
EHEC: enterohaemorrhagic escherichia coli
EIA: enzyme immunoassay
EMR: electronic medical records
EPEC: enteropathogenic escherichia coli
GBA: municipal administration
GP: general practitioner
IBD: inflammatory bowel disease
IBS: irritable bowel syndrome
ICPC: international classification of primary care
ICS: Immunochromatographic strip
IID: infectious intestinal disease
IRB: institutional review board
NZA: Dutch health authorities
PCR: polymerase chain reaction
STEC: Shiga toxin-producing Escherichia coli
TFT: triple faeces test
UGPN: Utrecht general practitioner network
XLD: xylose-lysine-deoxycholate
