# Supplementary material for: Design of the PROUD study: PCR faeces testing in outpatients with diarrhoea
Source: BMC Infect Dis. 2016 Jan 30;16:39. doi: 10.1186/s12879-016-1371-z (PMC4736251; doi:10.1186/s12879-016-1371-z)
Supplement: Supplementary file 1 — Secondary objectives of the PROUD study. (DOCX 18 kb) [file 12879_2016_1371_MOESM1_ESM.docx]

### Additional file 1. Secondary objectives of the PROUD study.

Two secondary objectives are studied within the PROUD study: i) guideline adherence; ii) role of IID in Irritable Bowel Syndrome (IBS) development.

#### Guideline adherence

Objective: To determine the proportion of patient referred for faeces testing in compliance with the guideline “acute diarrhoea” from the Dutch College of General Practitioners from 2007 before and after PCR introduction.

Measurements: For patients with a microbiological faeces test, the EMR charts of the related IID episode are extracted together with the clinical patient data (as outlined in the “measurements” section).

Outcome measure: As defined by the guideline “acute diarrhoea” from the Dutch College of General Practitioners [1], compliance is defined for bacterial testing when a patient suspected of IID is either (a) severely ill; (b) immunocompromised; (c) at increased risk for transmission; (d) diarrhoea duration of 10 or more days. Faeces testing for parasitic enteropathogens is only recommended in case diarrhoea duration of 10 or more days and testing for enteropathogenic viruses is not recommended.

Analysis: The difference in proportion of patients that are tested according to the Dutch GP’s guideline is compared in both the before and after periods (p≤0.05, two-tailed). Subsequently, an interrupted time series analysis is performed and adjustment made for other variables.

#### Role of IID in Irritable Bowel Syndrome (IBS) development

Objective: To determine the influence of relevant comorbidities and specific enteropathogens on the development of IBS after an episode of infectious intestinal disease.

Measurements: For all patients in the microbiological study novel diagnosis of IBS (ICPC D93) are extracted from the EMR one year after consultation of the GP with suspected IID. Detected relevant enteropathogens (bacterial, parasitic and viral) are detected by PCR testing (table1). Clinical patient data (as outlined in the “measurements” section) and known risk factors (e.g. psychosocial) for IBS development are gathered from the UGPN.

Outcome measure: Novel diagnosis of physician diagnosed IBS one year after infection with a relevant enteropathogen.

Analysis: Multivariable odds ratios are calculated for the contribution of infection with an enteropathogen (viral, bacterial and parasitic) additionally to known risk factors for the development of IBS using logistic regression.

1. Belo J, Bos M, Brühl P, Lemmen W, Pijpers M, Van der Donk M, Burgers JS, Bouma M, Loogman M: **NHG-Standaard Acute diarree**. 2014, **57**:462–71.
