# Supplementary material for: Design of the PROUD study: PCR faeces testing in outpatients with diarrhoea
Source: BMC Infect Dis. 2016 Jan 30;16:39. doi: 10.1186/s12879-016-1371-z (PMC4736251; doi:10.1186/s12879-016-1371-z)
Supplement: Supplementary file 2 — Measurements of basic patient characteristics and comorbidities coded with International Classification of Primary Care (ICPC). (DOCX 16 kb) [file 12879_2016_1371_MOESM2_ESM.docx]

### Additional file 2. Measurements of basic patient characteristics and comorbidities coded with International Classification of Primary Care (ICPC).

| **Variable** | **Variable type** | **ICPC code/values** |
| --- | --- | --- |
| ***Patient characteristics*** |  |  |
| Age | - | Date of birth |
| Gender | - | M/F |
| Socio-economic status | - | Zip code |
| ***Comorbidities*** |  |  |
| Malignant neoplasm bronchus / lung | Comorb. General | R84 |
| Other malignant neoplasm tr. resp. | Comorb. General | R85 |
| Chronic bronchitis / bronchiectasis | Comorb. General | R91 |
| Emphysema / COPD | Comorb. General | R95 |
| Asthma | Comorb. General | R96 |
| Angina pectoris | Comorb. General | K74 |
| Acute myocardial infarction | Comorb. General | K75 |
| Other / chronic ischemic heart disease | Comorb. General | K76 |
| Congestive heart failure | Comorb. General | K77 |
| Atrial fibrillation | Comorb. General | K78 |
| Cor pulmonale | Comorb. General | K82 |
| Non- rheumatic valve disease | Comorb. General | K83 |
| Other heart diseases | Comorb. General | K84 |
| Diabetes mellitus | Comorb. General | T90 |
| Dementia | Comorb. General | P70 |
| CVA | Comorb. General | K90 |
| Chronic alcohol abuse | Comorb. General | P15 |
| Irritable bowel syndrome | Comorb. General Intestinal | D93 |
| Chronic enteritis/colitis ulcerosa | Comorb. General Intestinal | D94 |
| Diverticulosis/diverticulitis | Comorb. General Intestinal | D92 |
| Other disease digestive organs | Comorb. General Intestinal | D99 |
| Hodgkin's lymphoma and other malignant lymphomas | Comorb. Immunocompromised | B72 |
| Leukaemia | Comorb. Immunocompromised | B73 |
| Other malignant tumours in blood or blood supplying organs | Comorb. Immunocompromised | B74 |
| Traumatic spleen rupture | Comorb. Immunocompromised | B76 |
| HIV-infection/AIDS/ARC | Comorb. Immunocompromised | B90 |
| Glomerulonephritis/nephrosis | Comorb. Immunocompromised | U88 |
| Other diseases of the urinary tract | Comorb. Immunocompromised | U99 |
| Renal function disorder/renal insufficiency | Comorb. Immunocompromised | U99.01 |
| Nephrosclerosis/hypoplastic kidney e.c.i. | Comorb. Immunocompromised | U99.02 |
| Obstructive and reflux-uropathy/hydronephrosis | Comorb. Immunocompromised | U99.03 |
| Other endocrine/metabolic/food related diseases | Comorb. Immunocompromised | T99 |
| Immunodeficiency | Comorb. Immunocompromised | T99.01 |
| Thyroiditis | Comorb. Immunocompromised | T99.02 |
| Cushing’s syndrome | Comorb. Immunocompromised | T99.08 |
| Addison’s syndrome | Comorb. Immunocompromised | T99.09 |
| Cystic fibrosis | Comorb. Immunocompromised | T99.10 |
| Liver cirrhosis /other liver disease | Comorb. Immunocompromised | D97 |
| Fatigue | Various | A04 |
| Feeling ill | Various | A05 |
| ***Risk factors*** |  |  |
| Drugs for acid related disorders | Risk factors diarrhoea | A02 |
| Corticosteroids | Immune compromising medications | H02 |
| Chemotherapy | Immune compromising medications | L01 |
| Immunosuppressants | Immune compromising medications | L04 |
| MTX | Immune compromising medications | L04AX03 |
