# Supplementary material for: Design of the PROUD study: PCR faeces testing in outpatients with diarrhoea
Source: BMC Infect Dis. 2016 Jan 30;16:39. doi: 10.1186/s12879-016-1371-z (PMC4736251; doi:10.1186/s12879-016-1371-z)
Supplement: Supplementary file 3 — Inclusion list of medication coded with Anatomical Therapeutic Chemical (ATC) classification system. (DOCX 15 kb) [file 12879_2016_1371_MOESM3_ESM.docx]

### Additional file 3. Inclusion list of medication coded with Anatomical Therapeutic Chemical (ATC) classification system.

| **ATC Description** | **Medication category** | **ATC code** |
| --- | --- | --- |
| Intestinal antispasmodics | Symptomatic medication | A03A |
| Anti-emetics | Symptomatic medication | A04A |
| Propulsives | Symptomatic medication | A03F |
| Probiotic antidiarrhoeals | Symptomatic medication | A07F |
| Antipropulsives | Symptomatic medication | A07D |
| Intestinal adsorbents | Symptomatic medication | A07B |
| Other drugs for acid related disorders | Symptomatic medication | A02X |
| Intestinal anti-infectives | Symptomatic medication | A07A |
| Drugs for acid related disorders | Symptomatic medication | A02 |
| ORS | Symptomatic medication | A07CA |
| Loperamide | Symptomatic medication | A07DA03, A07DA05, A07DA53 |
| Carbo adsorbens | Symptomatic medication | A07BA01, A07BA51 |
| Bismutsubsalicylaat | Symptomatic medication | A02BX05, A02BD08 |
| Azitromycine | Curative medication | J01FA10 |
| Ciprofloxacine | Curative medication | J01MA02 |
| Cotrimoxazol | Curative medication | J01EE01 |
| Metronidazol | Curative medication | A01AB17, J01XD01, P01AB01 |
| Erytromycine | Curative medication | J01FA01 |
| TMP-SMZ | Curative medication | J01EE01 |
| Doxycycline | Curative medication | A01AB22, J01AA02 |
| Vancomycine | Curative medication | A07AA09, J01XA01 |
| Teicoplanin | Curative medication | J01XA02 |
| Fidaxomicine | Curative medication | A07AA12 |
| Clioquinol | Curative medication | P01AA52, P01AA02 |
| Paromomycine | Curative medication | A07AA06 |
