# Supplementary material for: Design of the PROUD study: PCR faeces testing in outpatients with diarrhoea
Source: BMC Infect Dis. 2016 Jan 30;16:39. doi: 10.1186/s12879-016-1371-z (PMC4736251; doi:10.1186/s12879-016-1371-z)
Supplement: Supplementary file 4 — PCR laboratory procedures in microbiological study. (DOCX 17 kb) [file 12879_2016_1371_MOESM4_ESM.docx]

### Additional file 4. PCR laboratory procedures in microbiological study.

#### Viral enteropathogens

Faecal specimens (approximately 100mg or 100ul of fluid faeces) are added to 1ml of STAR buffer (Roche Diagnostics, Penzberg, Germany), and vortexed for complete homogenization. Thereafter, 100ul of Chloroform is added and the sample vortexed prior to a 1 minute centrifugation at 17,000 rpm. Subsequently, 200ul supernatant is first spiked during this first step of lysis and before DNA extraction with Phocine HerpesVirus and Murine Encephalomyocarditis Virus, serving as an internal process control (IPC) for DNA and RNA extraction and PCR inhibition, respectively. This process is followed by automated total nucleic acid extraction using the MagnaPure LC instrument and the Total Nucleic Acid Extraction kit (Roche, Penzeberg, Germany). Purified nucleic acid is eluted in 100ul elution buffer. Diagnostic Realtime PCR assays are performed for pan-Adenovirus and for Adenovirus type 40/41 specifically, as well as for the RNA viruses of interest (Rotavirus, Norovirus GI and GII, Astrovirus and Sapovirus), using an ABI 7500 realtime PCR system (Lifetechnology, Foster City USA). Positive and negative controls are included in each run. An IPC is present to monitor adequate performance of the entire laboratory procedure from extraction up the amplification and detection. Every IPC is spiked into each clinical sample prior to extraction. A result is considered to be valid if the IPC meets the pre-set acceptance criteria, meaning that the Cycle Threshold (Ct) value of the IPC falls within a ±2SD range from the mean Ct value that has been defined for this control in clinical samples. Samples are considered positive for a specific pathogen if the Ct value is <45 and the IPC meets the pre-set acceptance criteria for optimal amplification. Samples are considered negative if the Ct value exceeds 45 and the IPC does meets the pre-set acceptance criteria for optimal amplification.

#### Bacterial and parasitic enteropathogens

DNA isolation for PCR based detection of bacterial and parasitic pathogens approximately 100mg of each faecal sample are added to 1ml of lysis buffer (Cobas PCR Media [CMPNT GuaHCL REAGENT], Roche Molecular Systems Inc., Branchburg, USA). After homogenization (vortexing for approximately 15 seconds) the solution is frozen at -80C° for a minimum of 30 minutes. Just prior to DNA isolation frozen samples are heated at 95C° for 15 minutes in order to release the DNA on non-lysed cells and to inactivate DNAses. Samples are spiked with Phocine HerpesVirus during this first step of lysis, serving as an IPC for DNA extraction and PCR inhibition. DNA is then extracted from a 100ul aliquot of this sample on MagNA Pure96 (Roche), using the Roche MagNA Pure96 and Viral NA Small Volume Kit with the Viral NA Universal SV extraction protocol according to the manufacturer’s instructions. Final DNA elution is in 100ul, and 2ul of these elutes is used as input for all bacterial and protozoan pathogen PCR reactions. Positive and negative controls are included in each PCR run. PCRs are performed using a LightCycler 480 II (Roche) and are run 45 cycles. LightCycler 480 Probes Master (Roche) is used for the bacterial and protozoan pathogens specific PCR and reactions are performed in a total reaction volume of 15ul (R-Biopharm kits: 12ul mix + 3ul DNA, TIB MIOBIOL kits: 13ul Mix + 2ul DNA) in 384 well plates on the LightCycler 480. PCR reactions are performed as multiplex PCR reactions according tailored manufacturer’s conditions (adjusted the total reaction volume only) using the LightMix Modular Gastro Parasites and Gastro Bacteria kits (TIB Molbiol, GmbH Berlin, Germany) and RIDA®GENE Clostridium difficile Toxin A/B and E.Coli Stool Panel 1 kits(R-Biopharm AG, Darmstadt, Germany) for multiplex PCR. A result is considered to be valid if the IPC meets the pre-set acceptance criteria, meaning that the Cycle Threshold (Ct) value of the IPC falls within a ±2SD range from the mean Ct value that has been defined for this control in clinical samples. Samples are considered positive for a specific pathogen if the Ct value is <45 and the IPC meets the pre-set acceptance criteria for optimal amplification. Samples are considered negative if the Ct value exceeds 45 and the IPC does meets the pre-set acceptance criteria for optimal amplification. All PCR curves and the database are subsequently double-read to prevent interpretation and data-entry errors.
