# Supplementary material for: Design of the PROUD study: PCR faeces testing in outpatients with diarrhoea
Source: BMC Infect Dis. 2016 Jan 30;16:39. doi: 10.1186/s12879-016-1371-z (PMC4736251; doi:10.1186/s12879-016-1371-z)
Supplement: Supplementary file 5 — Expected 95 % CIs for enteropathogen proportions in a 1-year microbiological study. (DOCX 24 kb) [file 12879_2016_1371_MOESM5_ESM.docx]

### Additional file 5. Expected 95% CIs for enteropathogen proportions in a 1-year microbiological study.

| **Microorganism** | **Reported proportion (%)** | **95% CI width* for min/max expected sample size** (%)** |
| --- | --- | --- |
| *Campylobacter* spp. | 10.4 [1] | ±2.5 / ±1.6 |
| *Clostridium difficile* | 2.1 [2] | ±1.2 / ±0.8 |
| *Salmonella* spp. | 3.9 [1] | ±1.6 / ±1.0 |
| *Shigella* spp. | 0.1 [1] | ±0.3 / ±0.2 |
| *Yersinia* spp. | 0.7 [1] | ±0.7 / ±0.4 |
| Adenovirus | 2.2 [1] | ±1.2 / ±0.8 |
| Norovirus | 5.1 [1] | ±1.8 / ±1.2 |
| Rotavirus | 5.3 [1] | ±1.9 / ±1.2 |
| Astrovirus | 1.5 [1] | ±1.0 / ±0.6 |
| *Cryptosporidium* spp. | 2.1 [1] | ±1.2 / ±0.8 |
| *Entamoeba* spp*.* | 0.9 [1] | ±0.8 / ±0.5 |
| *Giardia lamblia* | 5.4 [1] | ±1.9 / ±1.2 |
| *Dientamoeba fragilis* | 10.3 [1] | ±2.5 / ±1.6 |
| *Blastocystis hominis* | 21.7 [1] | ±3.4 / ±2.2 |

*Expected precision of enteropathogen proportions in microbiological study, measured as the 95% confidence interval (CI) widths of reported enteropathogen proportions (based on [1, 2]).

** Expected minimum (n=554) and maximum (n=1,385) samples size of a 1-year microbiological study (based on [3, 4]).

1. de Wit MA, Koopmans MP, Kortbeek LM, van Leeuwen NJ, Vinjé J, van Duynhoven YT: **Etiology of gastroenteritis in sentinel general practices in the netherlands.** *Clin Infect Dis* 2001, **33**:280–8.

2. Wilcox MH, Mooney L, Bendall R, Settle CD, Fawley WN: **A case-control study of community-associated Clostridium difficile infection.** *J Antimicrob Chemother* 2008, **62**:388–96.

3. De Wit MA, Koopmans MP, Kortbeek LM, Wannet WJ, Vinjé J, van Leusden F, Bartelds AI, van Duynhoven YT: **Sensor, a population-based cohort study on gastroenteritis in the Netherlands: incidence and etiology.** *Am J Epidemiol* 2001, **154**:666–74.

4. Van den Brandhof WE, Bartelds AIM, Koopmans MPG, van Duynhoven YTHP: **General practitioner practices in requesting laboratory tests for patients with gastroenteritis in the Netherlands, 2001-2002.** *BMC Fam Pract* 2006, **7**:56.
